# Causes and Consequences of a Variant Strain of *Phaeobacter inhibens* With Reduced Competition

**DOI:** 10.3389/fmicb.2018.02601

**Published:** 2018-11-02

**Authors:** Marwan E. Majzoub, Kerensa McElroy, Michael Maczka, Torsten Thomas, Suhelen Egan

**Affiliations:** ^1^Centre for Marine Bio-Innovation, School of Biotechnology and Biomolecular Sciences, The University of New South Wales, Sydney, NSW, Australia; ^2^Agriculture and Food, Commonwealth Scientific and Industrial Research Organisation, Canberra, ACT, Australia; ^3^Institute of Organic Chemistry, Technische Universität Braunschweig, Braunschweig, Germany; ^4^Centre for Marine Bio-Innovation, School of Biological, Earth and Environmental Sciences, The University of New South Wales, Sydney, NSW, Australia

**Keywords:** marine bacteria, biofilm, roseobacter group, *Phaeobacter inhibens*, competition, microbial interactions, phenotypic variation, genomics

## Abstract

*Phaeobacter inhibens* 2.10 is an effective biofilm former and colonizer of marine surfaces and has the ability to outcompete other microbiota. During biofilm dispersal *P. inhibens* 2.10 produces heritable phenotypic variants, including those that have a reduced ability to inhibit the co-occurring bacterium *Pseudoalteromonas tunicata*. However, the genetic changes that underpin the phenotypic variation and what the ecological consequences are for variants within the population are unclear. To answer these questions we sequenced the genomes of strain NCV12a1, a biofilm variant of *P. inhibens* 2.10 with reduced inhibitory activity and the *P. inhibens* 2.10 WT parental strain. Genome wide analysis revealed point mutations in genes involved in synthesis of the antibacterial compound tropodithietic acid (TDA) and indirectly in extracellular polymeric substances (EPS) production. However, confocal laser scanning microscopy analyses found little differences in biofilm growth between *P. inhibens* 2.10 WT (parental) and NCV12a1. *P. inhibens* NCV12a1 was also not outcompeted in co-cultured biofilms with *P. tunicata*, despite its reduced inhibitory activity, rather these biofilms were thicker than those produced when the WT strain was co-cultured with *P. tunicata*. Notably, dispersal populations from biofilms of *P. inhibens* NCV12a1 had a higher proportion of WT-like morphotypes when co-cultured with *P. tunicata*. These observations may explain why the otherwise non-inhibiting variant persists in the presence of a natural competitor, adding to our understanding of the relative importance of genetic diversification in microbial biofilms.

## Introduction

Natural microbial biofilms are complex, highly structured communities enclosed within a self-produced matrix and are ubiquitous on all surfaces within aqueous environments (Davey and O’Toole, 2000; Dang et al., 2008). Laboratory-based studies have shown that biofilm development often follows distinct stages including: reversible attachment, irreversible attachment, biofilm development, biofilm maturation, and dispersal (Stoodley et al., 2002). A hallmark of biofilm dispersal is the generation of phenotypic variants that are heritable and distinct in characteristics from parental cells. This phenomenon of dispersal variation has now been observed for a number of bacterial taxa, including *Pseudomonas aeruginosa* (Webb et al., 2004; Kirisits et al., 2005; Luján et al., 2011), *Pseudomonas fluorescens* (Sanchez-Contreras et al., 2002; Sauer et al., 2002), *Pseudomonas putida* (Hansen et al., 2007), *Serratia marcescens* (Koh et al., 2007), *Pseudoalteromonas tunicata* (Mai-Prochnow et al., 2006), *Vibrio cholerae* (Matz et al., 2005), and *Phaeobacter inhibens* 2.10 (Lutz et al., 2016), where characteristics such as competitive fitness, colony morphology, colonization ability, and growth vary between individuals of the dispersing population. It has been proposed that the generation of heritable variants provides a mechanism for microbial populations to rapidly respond to changing environmental conditions (Aertsen and Michiels, 2004; Boles et al., 2004). Interestingly, recent work on mixed communities has shown that interspecific interactions (both competitive and co-operative) strongly influence the degree of phenotypic variation of dispersal populations (Lee et al., 2016; Lutz et al., 2016).

*Phaeobacter inhibens* 2.10 (formerly *Phaeobacter gallaeciensis* 2.10) is a dark pigmented member of the roseobacter group (Martens et al., 2006). This group of marine bacteria are widespread (Buchan et al., 2005; Wagner-Döbler and Biebl, 2006; Brinkhoff et al., 2008) and often abundantly observed in coastal and eutrophic zones (Buchan et al., 2014; Gifford et al., 2014; Wemheuer et al., 2015) and are effective colonizers of algal surfaces, such as the green macroalga *Ulva australis*, where they form part of the epiphytic biofilm community (Rao et al., 2005, 2010). Biofilm growth and dispersal of *P. inhibens* 2.10 result in the generation of heritable morphotypic variants that are white in color and are defective in their ability to inhibit the growth of the bacterium *P. tunicata* (Lutz et al., 2016). This genetic variation may be a result of neutral mutations and genetic drift with selection playing a role under certain conditions. For example, when *P. inhibens* 2.10 was co-cultured with *P. tunicata* the proportion of white, non-inhibiting variants in the population was significantly reduced compared to that observed for mono-cultures of the strains, demonstrating that biological interactions, such as the presence of a competitor, can influence (possibly via selection) phenotypic variation in biofilms. However, which genetic changes underpin the phenotypic variation, and what the ecological consequences are for individual variants within the population, remained unclear. To address these two questions, we determined the genome sequence of *P. inhibens* 2.10 WT (also referred to as the parental strain) and a non-inhibiting variant strain of *P. inhibens* 2.10 (designated NCV12a1) and compared them to each other and to the published *P. inhibens* 2.10 genome. We then investigated how strain NCV12a1 competed within mixed biofilms to understand if this genetic variation alters ecological interactions.

## Materials and Methods

### Bacterial Strains and Culture Conditions

*Phaeobacter inhibens* 2.10 WT (wild type) strain (Thole et al., 2012), *P. inhibens* NCV12a1 [non-inhibiting biofilm variant of *P. inhibens* obtained from (Lutz et al., 2016)], and *P. tunicata* D2 (Holmström et al., 1998) were routinely cultured in complex Marine Broth (Difco 2216 marine broth 37.4 g l^−1^) or Marine Agar (MA) (marine broth with 1.5% (w/v) Oxoid bacteriological agar). Bacterial strains were grown at 25°C with constant agitation of broth cultures at a speed of 180 rpm. For long-term storage each bacterial strain was kept at −80°C in 20% glycerol.

### Genome Sequencing Analysis

The genomic DNA from *P. inhibens* 2.10 parental and NCV12a1 strains was extracted using a potassium xanthogenate-sodium dodecyl sulfate DNA extraction method (Tillett and Neilan, 2000). Both strains were shotgun sequenced using a Nextera XT library preparation and Illumina MiSeq sequencing technology with a 2 bp × 300 bp chemistry at the Ramaciotti Centre for Genomics (RCG) at the UNSW Sydney. Raw sequence reads were first aligned to a reference genome (GenBank accession number CP002972; Thole et al., 2012) with Novoalign v3.02.12^1^, using the options -softclip 40 and -ILQ _QC and default values for all other parameters. No trimming or filtering was performed before alignment.

Following alignment, duplicate reads were identified using Picard tools v1.126’s FixMateInformation and MarkDuplicates^2^. Genetic variants were then called for all samples using the GATK v3.3-0-g37228af pipeline (McKenna et al., 2010) as follows: indels were realigned with RealignerTargetCreator and IndelRealigner, with dcov set to 1000 (downsampling); HaplotypeCaller was used in GVCF mode to identify candidate variants per sample with ploidy set to 1; followed by genotyping across all samples simultaneously using GenotypeGVCFs with -max_alternate_alleles set to 10 and -dcov set to 1000.

Genetic variants were manually confirmed by examining the realigned read alignments in IGV v2.3.55 (Robinson et al., 2011) and all were judged to be accurate [i.e., number of reads (coverage depth) greater than 10, no obvious bias in read position, strand, base quality, or mapping quality]. Annotations were assigned using SnpEff v4.1b (Cingolani et al., 2012) against the reference sequence database GCA_000154745.2.22. In reporting results, only the first annotation for each variant was retained. The sequence data was submitted to the BioSample database under accession numbers SAMN08473218 and SAMN07251612.

### Extraction and Detection of Tropodithietic Acid (TDA)

Tropodithietic acid (TDA) production was analyzed by high-performance liquid chromatography (HLPC). Briefly, *P. inhibens* 2.10 WT and NCV12a1 strains (70 ml) were incubated in MB for 120 h at 25°C on a rotary shaker (120 rpm). Cells were removed first by centrifugation (6,000 × *g*, 15 min, 4°C) and then by filtration through a 0.22-μm-pore-size mixed-cellulose-ester membrane, resulting in cell-free supernatant. The pH of the supernatant was adjusted to 3.0 with 2 M HCl, followed by extraction with 50 ml of ethyl acetate repeated twice. The vacuum-dried ethyl acetate extract was dissolved in 1 ml of acetonitrile and analyzed on a high-pressure liquid chromatography (HPLC) system (Thermo Fisher Scientific, Waltham, MA, United States) equipped with Thermo Fisher LTQ XL mass detector (LC-MS) in ESI-negative mode. Samples were separated on an Agilent Zorbax Eclipse Plus C_18_ column (Macherey-Nagel, Düren, Germany) by using an acetonitrile-water gradient system containing 0.25% formic acid, started with 5% acetonitrile, which was increased linearly to 95% in 8 min. For the generation of the standard curves, pure TDA dissolved in a 1:1 H_2_O/Acetonitrile mixture at 1 mM and a standard set containing concentrations (1, 2.5, 5, 10, 25, and 50 μM) were used. For each assay, an internal standard curve was created in triplicates by calculating the ratio of TDA concentrations by integrating the TDA peak. Only standard curves with a correlation coefficient (*r*^2^) of >0.95 were used for quantification. The detection limit of this assay was less than 2.5 μM. The concentration of TDA produced was reported as nanomolar.

### Quantification of Exopolysaccharide Production

*Phaeobacter inhibens* 2.10 WT and NCV12a1 strains were grown shaking in culture flasks as described above at 25°C for 24, 48, and 72 h and EPS production (both attached to bacteria and present in culture flask) was quantified as described previously (Tapia et al., 2009) with minor modifications. Briefly, 8 ml of 1 M of NaOH was added to 20 ml of cultures for each time-point, incubated at room temperature for 3 h to extract EPS. The cell suspension was then centrifuged for 30 min at 16,800 × *g* and 4°C. The supernatant containing soluble EPS was then filtered through a 0.2 μm filter. 1.5 volumes of 96% ice-cold ethanol were added and the mixture was placed at −20°C for 24 h to separate exopolysaccharides away from lipids. The precipitate was collected after centrifugation (16,800 × *g*) for 30 min at 4°C. EPS produced was quantified as EPS dry weight (g) per liter of culture. A total of two independent experiments with three independent measurements each were conducted. Mean and standard error was calculated using GraphPad Prism 6.03 (San Diego, CA, United States). An unpaired *t*-test was used to determine significance between independent experiments for either the WT or variant NCV12a1. Difference between independent experiments was not significant (i.e., *p* > 0.05) therefore all independent measurements for WT and variant NCV12a1 were combined and analyzed as one experiment (i.e., *n* = 6). Differences in EPS production between *P. inhibens* 2.10 WT and NCV12a1 variant strains were assessed using two-way ANOVA with Sidak’s multiple comparison testing.

### Quantification of Bacterial Cell Attachment and Batch Biofilm Formation

Cell attachment (measured after 2 h incubation) and batch biofilm biomass (after 24 h incubation) of *P. inhibens* 2.10 WT and NCV12a1 strains were quantified by the crystal violet staining assay as described previously (O’Toole and Kolter, 1998). Briefly, to assess for attachment and batch biofilm growth, cultures were inoculated into 96-well and 24-well sterile polystyrene plates, respectively, and incubated at 25°C for 24 h with constant agitation at 60 rpm. Absorbance for each well measured at 550 nm using a Wallac Victor2 1420 Multilabel counter (PerkinElmer Inc.). A total of two independent experiments were conducted with six independent measurements for cell attachment and four independent measurements for batch biofilm formation. Mean and standard error were calculated using GraphPad Prism 6.03 (San Diego, CA, United States). An unpaired *t*-test was used to determine the significance between independent experiments for either the WT or variant NCV12a1. The difference between independent experiments was not significant (i.e., *p* > 0.05) therefore replicate measurements for WT and variant NCV12a1 from both experiments were combined and analyzed together (i.e., *n* = 12). The significance between *P. inhibens* WT and variant NCV12a1 was determined using a one-way ANOVA.

### Continuous Culture Flow Cell System Biofilms

*Phaeobacter inhibens* 2.10 WT, *P. inhibens* NCV12a1, and *P. tunicata* strains were labeled with either a red fluorescent protein (RFP) or a green fluorescent protein (GFP) color tag by transconjugation using the plasmid pCJS10R or pCJS10G (Rao et al., 2005), respectively. Resulting in the following strains *P. inhibens* 2.10 WT (RFP and GFP), *P. inhibens* NCV12a1 (RFP and GFP), and *P. tunicata* (GFP). Biofilms were cultivated in three-channel flow cells (channel dimensions, 1 mm × 4 mm × 40 mm) as described previously (Moller et al., 1998). The flow cells were continuously fed for 12 days with a marine minimal medium (MMM) (Neidhardt et al., 1974) using a peristaltic pump at a flow rate of 1.32 ml min^−1^. Each channel was injected with 0.5 ml of diluted overnight culture containing 1 × 10^8^ colony-forming units (CFU) ml^−1^ and allowed to settle for 1.5 h before turning on flow. Mixed-species competition biofilms were established as previously described (Lutz et al., 2016) by inoculating mixed cultures of *P. inhibens* 2.10 and *P. tunicata* in the ratio of 1:10. To assess morphotypic variation of dispersal populations, biofilm effluent was spread plated onto MA plates daily from 2 to 12 days of biofilm growth. Plates were incubated at 25°C for 3 days and thereafter colonies were counted and colony morphologies were described. For biofilms of strain *P. inhibens* NCV12a1 colonies were described as either “white” or “other” and the total percentage of each phenotypic variant type was calculated. A total of two independent experiments were conducted and data plotted using GraphPad Prism 6.03 (San Diego, CA, United States).

### Confocal Laser Scanning Microscopy (CSLM)

Biofilm development was assessed at 3, 7, 10, and 12 days of biofilm growth using a Leica TCS SP5 MP STED (Biomedical Imaging Facility UNSW, BMIF) confocal laser scanning microscope (CSLM). Five fields of view of each surface were randomly selected for 3D imaging for each flow cell channel. Each field of view covered 387.5 μm × 387.5 μm of surface area and Z-stacks (0.25 μm each) imaging was performed using the “LEICA” image software and default settings (Leica Application Suite Advanced Fluorescence [LAS AF]) to obtain a three-dimensional (3D) structure of the established biofilm. The excitation wavelengths for GFP and RFP were 488 and 561 nm, respectively. All images were captured using 40× objective (oil immersion, NA 1.25; Leica). The CSLM images were then analyzed with IMARIS (Bitplane Scientific Software Inc., 7.4.2) using the default parameters. Information including biovolume, surface area, and biofilm thickness were recorded. Total biovolume/surface area and thickness were plotted using PRISM 6.03 (GraphPad Software Inc.). A two-way ANOVA with repeated measures was performed to determine the significance between strains with Sidak’s multiple comparison tests to identify the differences in data obtained for biofilm development among strains at each time-point.

## Results and Discussion

### Genetic Mutations Identified in the *P. inhibens* NCV12a1 Variant Strain

Genome sequencing and comparative analysis was performed to identify the underlying genetic changes associated with the *P. inhibens* NCV12a1 variant strain. We detected two mutations (one insertion and one duplication) in the variant genome when it was mapped to the newly sequenced *P. inhibens* 2.10 WT parental genome. One of the mutations was in a region of the genome related to TDA synthesis and the second was in a gene related to cell wall recycling and linked to EPS production (Table 1), suggesting these functions are directly or indirectly related to the decrease in competitive fitness previously reported for this strain (Lutz et al., 2016). It is worth noting that an additional 21 nucleotide changes were found in both the *P. inhibens* NCV12a1 variant and the *P. inhibens* 2.10 WT parental strain when it was mapped to the original *P. inhibens* 2.10 reference genome (GenBank accession number CP002972; Thole et al., 2012), a list of those mutations is provided in the Supplementary Material (Supplementary Table S1). The *P. inhibens* 2.10 culture used in this study originated from the sequenced reference strain (Thole et al., 2012) and while these additional mutations may reflect natural genetic drift, selection during laboratory cultivation can’t be ruled out.

**Table 1:** Genetic variants detected in *P. inhibens* NCV12a1 genome compared to *P. inhibens* 2.10 WT parental.

Locus tag	Replicon	Position	Annotation	Gene	Predicted product/function [COG]	Variant	Effect
**TDA production**							
PGA2_239p0960	pPGA2_239	106349	Upstream_gene_variant	*tdaB*	Glutathione S-transferase [O]	c.-1_-1insT	Unknown
**EPS production**							
PGA2_c11200	Chromosome	1233726	Frameshift_variant	*anmK*	Anhydro-N-acetylmuramic acid kinase [M]	c.1163dupG	p.Glu389fs

*The column “Replicon” refers to whether the mutation was detected in the chromosome or the plasmids. The column “Position” refers to the position relative to the start of the replicon. The columns “Gene” and “Function” refer to the name and the predicted function of the homologous gene in the P. inhibens reference genome. Clusters of Orthologous Groups (COGs) functional categories: M, Cell wall/membrane/envelope biogenesis; O, Post-translational modification, protein turnover chaperones. The column designated “Variant” refers to the original nucleotide and the nucleotide mutation that was detected. Duplications (dup) or insertions (ins) are given relative to the start of the gene (c). The column designated “Effect” indicates the amino acid level changes that occur as a result of the variant mutation.*

*Phaeobacter inhibens* 2.10 and related strains such as *P. inhibens* DSM 17395 produce the antibacterial compound TDA that can inhibit or kill other bacteria (Brinkhoff et al., 2004; Bruhn et al., 2005; Porsby et al., 2008; Geng and Belas, 2010; Berger et al., 2011; Garcia et al., 2014). In *P. inhibens* DSM 17395 production of TDA is partly controlled in a cell-density dependent manner by acylated homoserine lactone (AHL) signaling molecules and by TDA itself (Geng and Belas, 2010, 2011; Berger et al., 2011). Recent studies further suggest that TDA has a much broader role in gene regulation and may act as a functional analog to AHL in *P. inhibens* DSM 17395 (Beyersmann et al., 2017). Loss of TDA production in *P. inhibens* NCV12a1 was confirmed by HPLC analysis, which detected a dominant peak (7.44 min) typical for pure TDA (Bruhn et al., 2005) in crude extracts of *P. inhibens* 2.10 WT, but not in *P. inhibens* NCV12a1 (Supplementary Figure S1).

In other roseobacter group organisms, including *P. inhibens* DSM 17395 and *Ruegeria* sp. strain TM1040 (formally *Silicibacter* sp.), the lack of TDA production has been linked to the loss of the plasmid encoding its synthesis (Geng et al., 2008; Brock et al., 2014); however, this was not the case for *P. inhibens* NCV12a1. Instead, a single base pair insertion was detected in the intergenic region of genes with homology to *tdaB* and *tdaC*, respectively (Table 1). These genes form part of a plasmid-encoded TDA biosynthetic cluster, which consists of the two operons *tdaAB* and *tdaCDE* (Geng and Belas, 2011). Expression of *tdaCDE* is under the control of the LyS-R type transcriptional regulator (LTTR) TdaA, which specifically binds to the *tdaC* promoter region. Closer inspection of this region in *P. inhibens* NCV12a1 found that the aforementioned insertion was located within the predicted LTTR binding site (i.e., TCAGGTTTTTTGAA at positions −223 to −211) of the *tdaC* promoter (Geng and Belas, 2011). Such a mutation may impact the ability of TdaA to bind to the promoter resulting in reduced *tdaCDE* expression and loss of TDA production.

**FIGURE 1:**
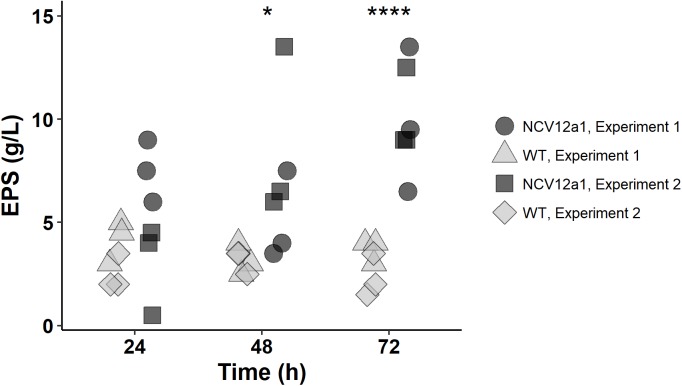
Amount of EPS produced by *P. inhibens* 2.10 wild-type (light gray symbols) and *P. inhibens* variant NCV12a1 (dark gray symbols) strains in MB liquid medium after 24, 48, and 72 h of incubation at 25°C. Plotted points indicate replicate measures (*n* = 3) for each individual experiment. As there was no statistical difference between experiment 1 and 2 (*P* > 0.05), a two-way ANOVA was performed on the data pooled from both experiments, significant differences were observed between *P. inhibens* 2.10 WT and *P. inhibens* 2.10 variant NCV12a1 after 48 h (*p* = 0.0281; ^∗^) and 72 h (*p* < 0.0001; ^∗∗∗∗^) of growth.

A second mutation was detected in a gene (PGA2_c11200) with homology to *anmK*, encoding a putative anhydro-N-acetylmuramic acid kinase. While the precise function of the *anmK* gene in *P. inhibens* is unknown, homologs have been shown in other bacteria to be involved in cell wall recycling (Uehara et al., 2005; Gisin et al., 2013) and affect alginate biosynthesis in *P. fluorescens* (Ertesvåg et al., 2017). Alginate is a main component of the extracellular polymeric substance (EPS) studied predominately in species of *Pseudomonas* where it plays an important role in enhancing adhesion to surfaces (Limoli et al., 2015). While alginate production has not been observed in *P. inhibens* 2.10, mutations in a homolog of *algA*, a precursor to alginate and EPS production show reduced biofilm formation in *P. inhibens* DSM17395 (Garcia et al., 2014). Given the role of EPS in biofilm formation we assessed if EPS production is impacted by the mutation in the *anmK* homolog by quantifying EPS for both *P. inhibens* 2.10 WT and *P. inhibens* NCV12a1 strains. We observed that after 3 days growth NCV12a1 produced more EPS compared to *P. inhibens* 2.10 WT (*p* < 0.0001) (Figure 1). EPS overproducing variants have also been observed for a number of other biofilm forming bacteria including *P. fluorescens* (Spiers et al., 2003), *P. aeruginosa* (de Regt et al., 2014), and *V. cholerae* (Yildiz and Schoolnik, 1999). The increase in EPS production is thought to allow these bacteria to persist in unfavorable conditions, including starvation, low temperature and pH, oxidative stress, elevated UV light, and exposure to toxins (Wai et al., 1998; Mizunoe et al., 1999; Yildiz and Schoolnik, 1999; Uhlich et al., 2006; White and Surette, 2006). Therefore overproduction of EPS by *P. inhibens* NCV12a1 could similarly act as a protective mechanism for the cells, indirectly compensating for the loss of TDA production.

### The Effect of Intraspecific and Interspecific Competition on Biofilm Development of *P. inhibens* NCV12a1

We next investigated how these observed genetic changes may impact intra- and interspecies competitiveness in biofilms comprised of *P. inhibens* NCV12a1 co-cultured with either the *P. inhibens* 2.10 WT or *P. tunicata* in a continuous flow cell system. *P. inhibens* 2.10 WT (RFP) *P. inhibens* variant NCV12a1 (GFP and RFP) and *P. tunicata* (GFP) labeled strains were inspected under a fluorescence microscopy (data not shown) indicating the labeling of cells was successful. Growth curves of both newly labeled strains and corresponding unlabelled strains confirmed that labeling did not impact cell growth (data not shown). When grown individually there were no significant differences in attachment and biofilm formation (*p* > 0.05) between *P. inhibens* NCV12a1 and WT strains (Supplementary Figure S2). Biofilm development was likewise similar for both strains across all days with the exception of day seven (Supplementary Figures S3a,b), where biofilm thickness was greater for NCV12a1 (*p* < 0.05) (Supplementary Figure S3b), which may be a reflection of the higher EPS production in this strain. In co-culture, both *P. inhibens* WT and NCV12a1 strains were present in equal proportions throughout the experiment indicating that there is no competitive advantage for *P. inhibens* NCV12a1 strain in an intraspecific biofilm setting (*p* > 0.05) (Supplementary Figures S4a,b). This result is somewhat surprising given that several studies have shown that “cheater” variants, which are defective in the production of public goods, such as antibiotics, signaling molecules, or siderophores, can outcompete wild-type populations due to energetic costs saving (Velicer, 2003; Griffin et al., 2004; Travisano and Velicer, 2004; Jiricny et al., 2010; Allison et al., 2014). Despite TDA production being a metabolic burden to other *P. inhibens* strains (Will et al., 2017) it is possible that under the conditions tested here, the loss of production of TDA did not generate a competitive energetic saving or that this was balanced out by the additional cost associated with, for example, the increased production of EPS. To test these hypotheses future studies should assess the overall energy balance requirements and costs associated with TDA and EPS of the variant and the wild type strains used here.

**FIGURE 2:**
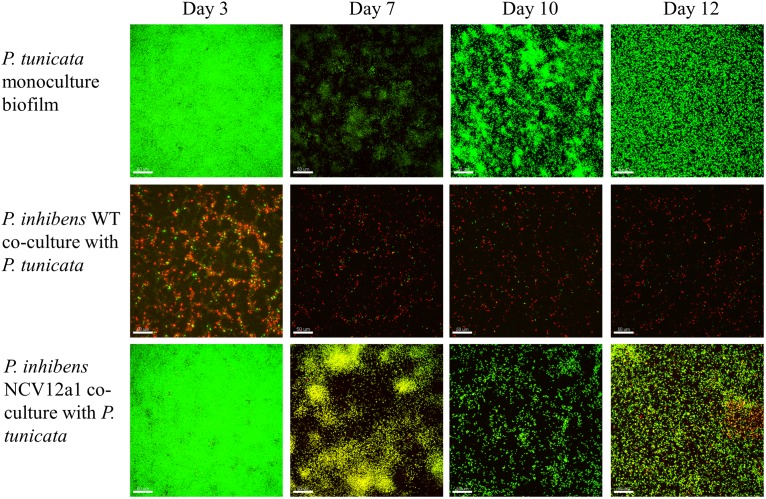
Confocal laser scanning microscope (CSLM) images of *P. inhibens* 2.10 parental WT (RFP-labeled) monoculture biofilm, *P. inhibens* variant NCV12a1 [RFP-labeled] monoculture biofilm, *P. tunicata* (GFP-labeled) monoculture biofilm, *P. inhibens* 2.10 parental WT (RFP-labeled) co-culture with *P. tunicata* (GFP-labeled), and *P. inhibens* variant NCV12a1 (RFP-labeled) co-culture with *P. tunicata* (GFP-labeled) biofilms grown in continuous flow cell systems at 3, 7, 10, and 12 days of biofilm development. The scale bar represents 50 μm.

**FIGURE 3:**
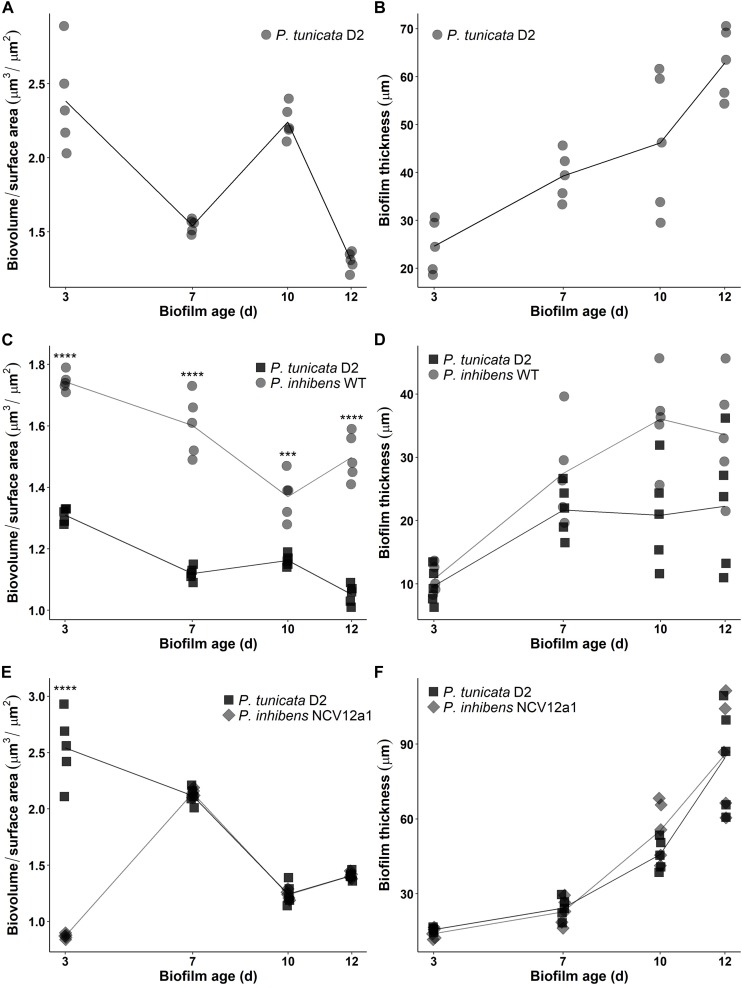
Time-series of biofilm development in continuous flow cell system for *P. tunicata* monoculture reported as biovolume over surface area (μm^3^/μm^2^) **(A)** and thickness (μm) **(B)**; *P. inhibens* 2.10 WT (circle symbol) co-cultured with *P. tunicata* (square symbol) reported as biovolume over surface area (μm^3^/μm^2^) **(C)** and thickness (μm) **(D)** and the *P. inhibens* NCV12a1 (diamond symbol) co-cultured with *P. tunicata* (square symbol) reported as biovolume over surface area (μm^3^/μm^2^) **(E)** and thickness (μm) **(F)** in a continuous flow cell system. Biofilms were observed on 3, 7, 10, and 12 days. Plotted points indicate replicate measures (*n* = 3) for each individual experiment. Lines represent the mean for each experiment. Statistical analysis was performed using two-way ANOVA. Significant differences for label **C** were observed after 3, 7, and 12 days of biofilm growth (*p* < 0.0001; ^∗∗∗∗^) and 10 days of growth (*p* = 0.0009; ^∗∗∗^). Statistical differences for label **E** was observed after 3 days of biofilm growth (*p* < 0.0001; ^∗∗∗∗^).

In addition to these within-species processes, naturally occurring biofilms also experience competitive interactions between populations or species, due to limited resources, such as nutrients and space (Egan et al., 2008; Wahl et al., 2012). Previous studies have used the epiphytic bacteria *P. inhibens* and *P. tunicata* as a model system to understand competitive interactions between co-occurring marine bacteria (Rao et al., 2005, 2006, 2007). These studies have shown that under all conditions tested *P. inhibens* outcompetes or reduces the numbers of *P. tunciata* when co-cultured in static biofilms on both artificial and living surfaces for short periods of time (Rao et al., 2005, 2006). We observed similar patterns of competition for the *P. inhibens* 2.10 WT strain and *P. tunicata* during early biofilm growth in flow cells, with *P. inhibens* 2.10 WT cells contributing to a greater proportion of the biovolume and thickness in co-cultured biofilms (Figures 2, 3), with significant differences observed in biovolume of each strain across all days (*p* < 0.05) (Figure 3C). In contrast, significantly more *P. tunicata* cells contributed to the biovolume of biofilms when co-cultured with *P. inhibens* NCV12a1 on day three (*p* < 0.0001) (Figure 3E). However, by day seven equal biovolumes and thickness were detected for both strains (*p* > 0.05) (Figures 3E,F) indicating that NCV12a1 was initially negatively impacted by interspecific competition, but then could nevertheless persist in the presence of *P. tunicata*. In addition, biofilms of *P. inhibens* NCV12a1 in co-culture with *P. tunicata* form much thicker biofilms than those formed between the WT strain and *P. tunicata* (Figures 3D,F). While the consequences for *P. inhibens* living in a thicker biofilm are unclear, enhanced biofilm formation has previously been shown for mixed species cultures, with beneficial effects such as increased resistance to antibiotics and grazers compared to their monoculture counterparts (e.g., Burmølle et al., 2006; Ren et al., 2015; Lee et al., 2016). Thus it is possible that in the absence of strong inhibition of *P. tunciata* by *P. inhibens* NCV12a1, the interaction between these strains shifts from being predominately competitive (as seen in WT co-cultures) to neutral or even synergistic.

Another possible explanation for the observed differences in interspecific competition of the WT and variant strains during biofilm development could be that TDA production is required for it to be an effective competitor during early stage biofilm development. However, once the biofilm matures these characteristics are either no longer required or secondary genetic mutations occur in the variant strain such as those that result in higher EPS production (and possibly increased biofilm thickness) or yet unidentified mutations. Such secondary mutations may then compensate for the original mutations, thereby preventing *P. inhibens* NCV12a1 from being completely out-competed by *P. tunciata*. While it is possible that both scenarios contribute to these observations, we did find a relatively low frequency (<2%) of WT-like morphotypic colonies arising from the biofilm effluent of *P. inhibens* NCV12a1 (Supplementary Figure S5). These WT-like variants appeared earlier (day 2 vs. day 3) and reached higher numbers (peaking at 4% on day 6) in the biofilms co-cultured with *P. tunicata* than in monoculture biofilms (Supplementary Figures S5a,b), suggesting that either the original mutation is being restored or additional compensatory mutations are generated.

In summary, biofilm growth of *P. inhibens* 2.10 results in the appearance of phenotypic variants harboring mutations that could affect the competitive ability of this strain, including mutations in genes affecting the synthesis of EPS and the antibacterial compound TDA. Biofilm formation and TDA production have been characterized as important ecological traits for members of the roseobacter group. Therefore their loss in natural populations may have profound consequences for the success of these bacteria. Nevertheless roseobacters remain abundant in many marine habitats. One explanation for this apparent paradox is provided by studies showing a reduced frequency of phenotypic variation in dispersal cells from mixed biofilms (Lee et al., 2016; Lutz et al., 2016), suggesting that the bacterial interactions occurring in natural environments may prohibit the proliferation of these genetic mutations outside of a laboratory setting. As the genetic changes investigated here were found to emerge under laboratory conditions further work will be required to establish their existence and significance in the natural environment. Moreover we found that despite *P. inhibens* NCV12a1’s reduced ability to inhibit other bacteria in plate assays, there was minimal consequence of this biofilm variant on intraspecific and interspecific competition during subsequent biofilm development in the laboratory. Future work will involve analyzing the genomes and phenotypes of additional *P. inhibens* 2.10 variants to determine if the mutations relating to TDA and EPS production detected for NCV12a1 is representative of the full spectrum of possible mutations that result in reduced competitive fitness under laboratory and environmental conditions.

## Author Contributions

MEM, TT, and SE designed the experiments. MEM, KM, MM, and TT performed the experiments and analyzed the data. MEM, KM, and SE designed and prepared the figures and tables and drafted the original manuscript. All authors contributed to discussion of results, critical revisions during the editing process, and approved the final manuscript draft for submission.

## Conflict of Interest Statement

The authors declare that the research was conducted in the absence of any commercial or financial relationships that could be construed as a potential conflict of interest.

## Footnotes

1 www.novocraft.com

2 http://broadinstitute.github.io/picard
