# Supplementary material for: Causes and Consequences of a Variant Strain of Phaeobacter inhibens With Reduced Competition
Source: Front Microbiol. 2018 Nov 2;9:2601. doi: 10.3389/fmicb.2018.02601 (PMC6224355; doi:10.3389/fmicb.2018.02601)
Supplement: Supplementary file 1 [file Data_Sheet_1.PDF]

Supplementary material for Majzoub *et al*:

Causes and consequences of a variant strain of *Phaeobacter inhibens* with reduced competition

Marwan Majzoub[1], Kerensa McElroy[2], Michael Maczka[3], Torsten Thomas[4] and Suhelen Egan[4#]

[1] Centre for Marine Bio-Innovation and School of Biotechnology and Biomolecular Sciences, The University of New South Wales, Sydney, New South Wales, Australia

[2] Agriculture and Food, Commonwealth Scientific and Industrial Research Organisation (CSIRO), Canberra, Australia

[3] Institute of Organic Chemistry, TU Braunschweig, Braunschweig, Germany

[4] Centre for Marine Bio-Innovation and School of Biological, Earth and Environmental Sciences, The University of New South Wales, Sydney, New South Wales, Australia

[#]Corresponding author

Assoc. Prof. Suhelen Egan

Centre for Marine Bio-Innovation

School of Biological, Earth and Environmental Sciences

The University of New South Wales, NSW 2052, Australia

Ph + 61 (0) 2 938 53467

Email: s.egan@unsw.edu.au

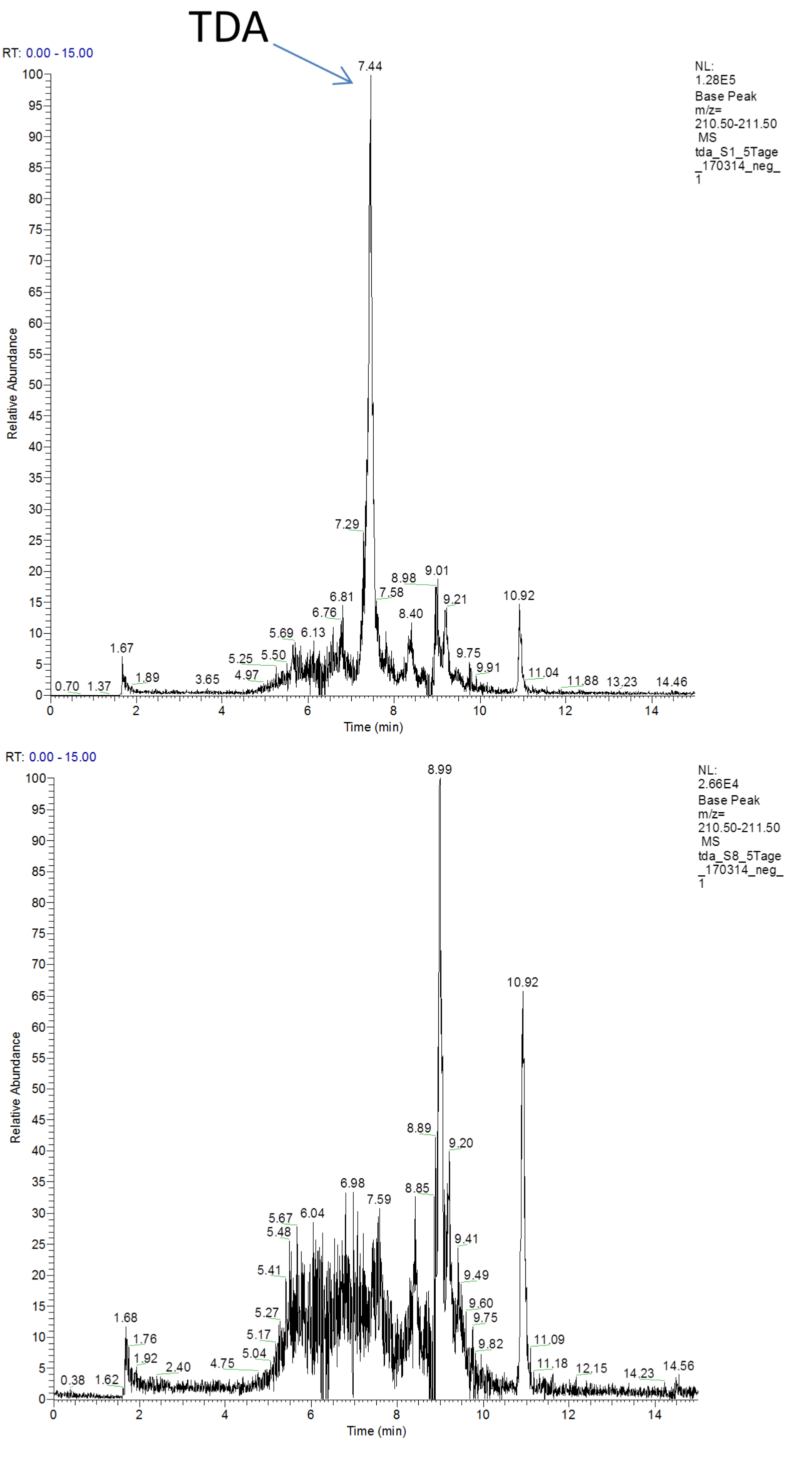

Figure S1: HPLC chromatogram of TDA obtained from *P. inhibens* 2.10 parental WT (a) and *P. inhibens* variant NCV12a1 (b) ethyl acetate extracts. The dominant peak at 7.44 mins corresponds to TDA (Bruhn et al., 2005).

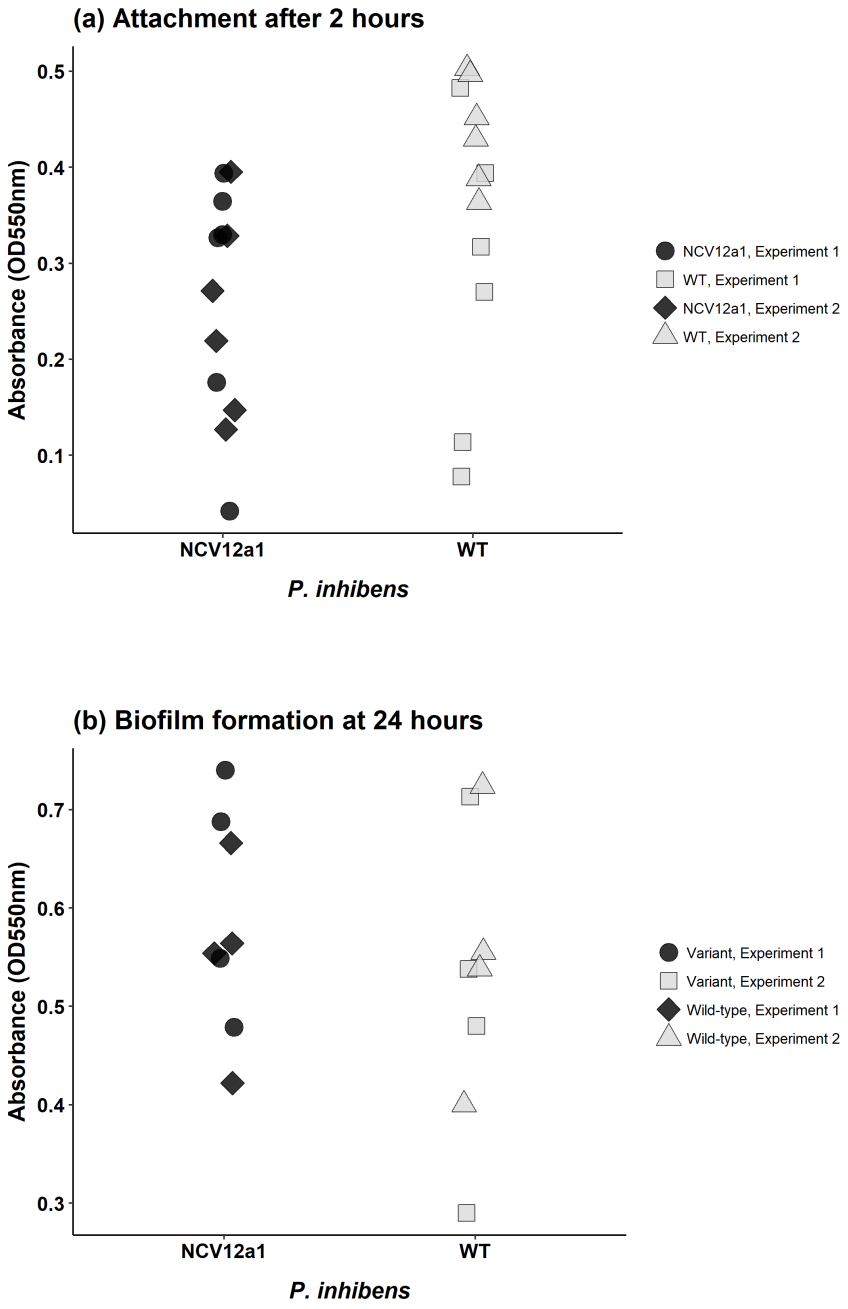

Figure S2: Surface attachment (a) and biofilm formation (b) by *P. inhibens* 2.10 WT and variant NCV12a1. Plotted points indicate replicate measures ((a) n=12, (b) n=8) for each individual experiment. The experiments for *P. inhibens* WT and NCV12a1 are independent. No significant differences were observed between *P. inhibens* WT and NCV12a1 ($p > 0.05$).

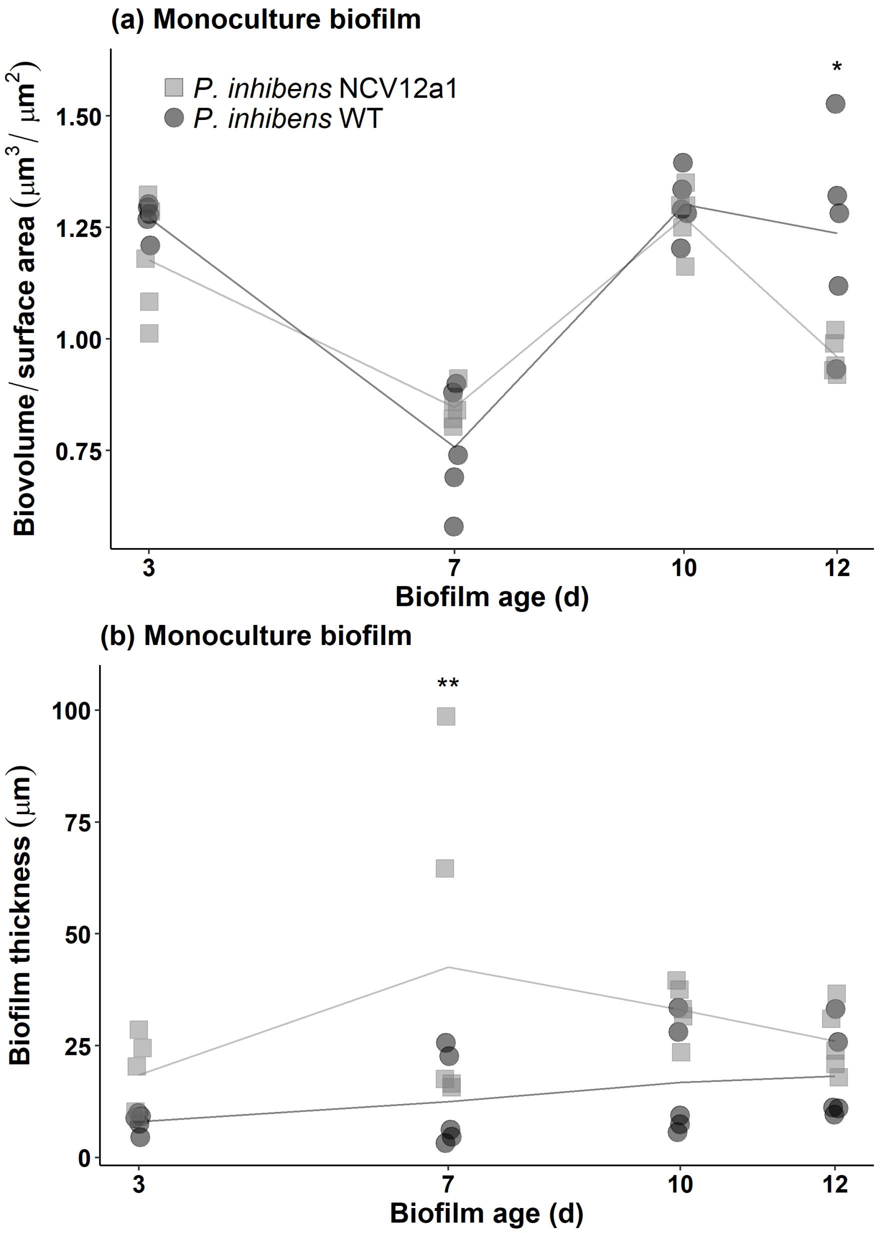

Figure S3: Time-series of *P. inhibens* 2.10 WT (circle symbol) and NCV12a1 (square symbol) monoculture biofilm development in continuous flow cell system reported as biovolume over surface area ($\mu m^3 / \mu m^2$) (a), and thickness (μm) (b). Biofilms were observed on 3, 7, 10 and 12 d. Plotted points indicate replicate measures (n=5) for each individual experiment. Lines represent the mean for each experiment. Statistical analysis was performed using two-way ANOVA. Significant differences for figure (a) was observed after 12 d of biofilm growth (p = 0.0337; *). Statistical differences for figure (b) was observed after 7 d of biofilm growth (p = 0.0084; **).

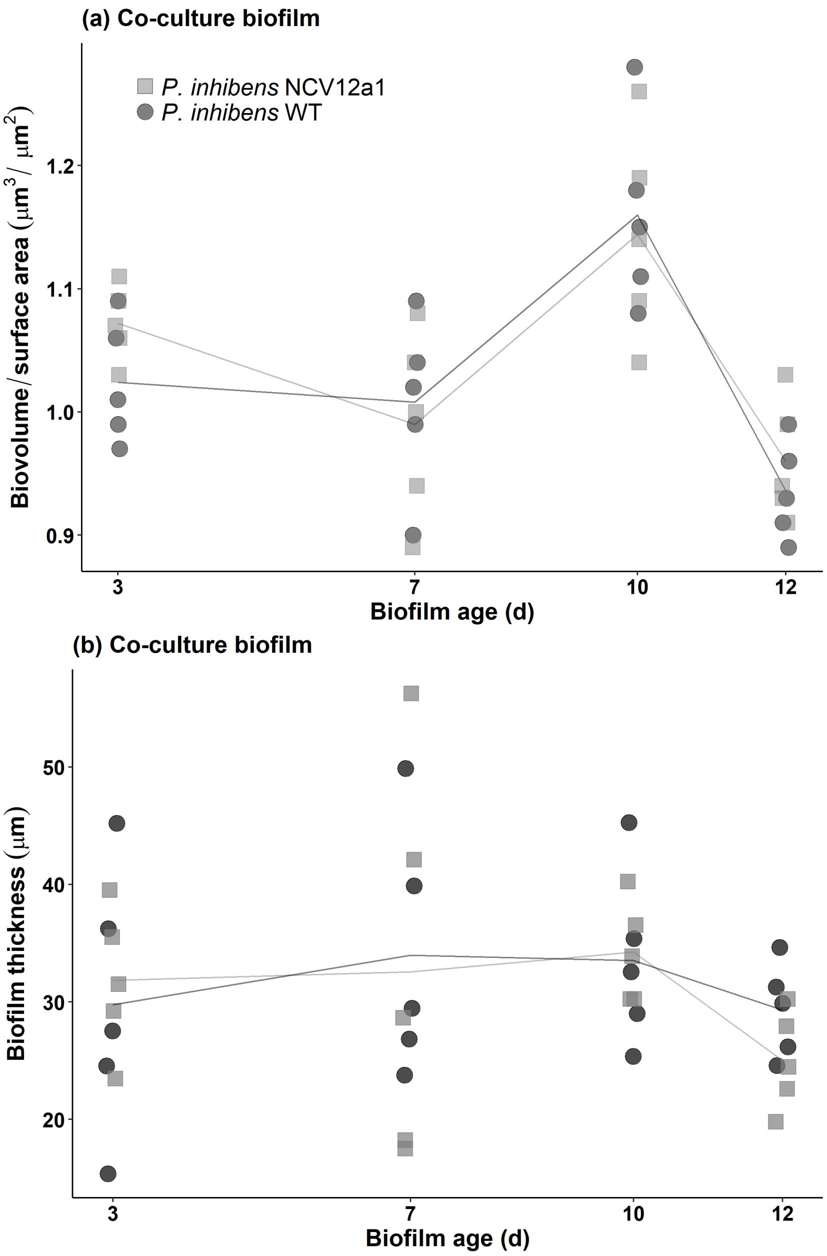

Figure S4: Time-series biofilm development showing the proportion of *P. inhibens* 2.10 WT (circle symbol) and NCV12a1 (square symbol) when grown as mixed-species biofilms in continuous flow cell system. Biofilms are reported as biovolume over surface area ($\mu m^3/ \mu m^2$) (a) and thickness (µm) (b). The established biofilms were observed on 3, 7, 10 and 12 d. Plotted points indicate replicate measures (n=5) for each individual experiment. Lines represent the mean for each experiment. No significant differences were observed at each time point.

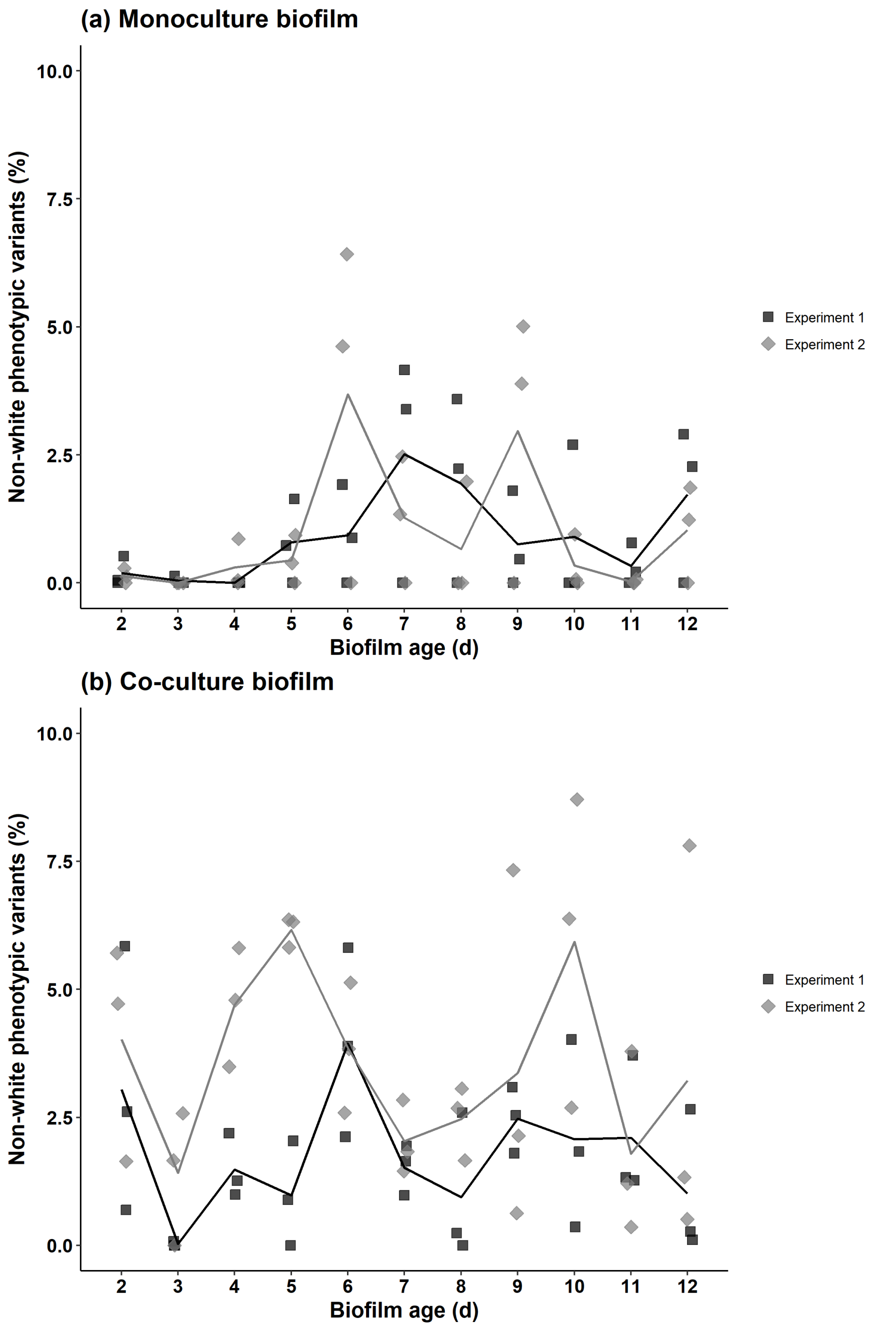

Figure S5: Phenotypic variation of *P. inhibens* NCV12a1 monoculture biofilm (a) and co-culture biofilm with *P. tunicata* (b) colonies over a period of 12 d within a continuous biofilm flow cell system. Viable effluent cells were collected each day for 12 d. Plotted points indicate the percentage (n=6) of *P. inhibens* variant NCV12a1 'other' colony variants obtained at each day of biofilm growth. Lines represent the mean for each experiment.

Table S1: Mutations detected in *P. inhibens* 2.10 WT parental genome compared to *P. inhibens* 2.10 reference genome. The column "Replicon" refers to whether the mutation was detected in the chromosome or the plasmids. The column "Position" refers to the position relative to the start of the replicon. The columns "Gene" and "Function" refer to the name and the predicted function of the homologous gene in the *P. inhibens* reference genome. Clusters of Orthologous Groups (COGs) functional categories: M: Cell wall/membrane/envelope biogenesis; L: Replication, recombination and repair; P: Inorganic ion transport and metabolism; R: General function prediction only; C: Energy production and conversion; D: Cell cycle control/cell division/chromosome partitioning; N: Cell motility; T: Signal transduction mechanisms; S: Function unknown; O: Post-translational modification, protein turnover chaperones. The column designated "Variant" refers to the original nucleotide and the nucleotide mutation that was detected. SNPs, deletions (del), duplications (dup) or insertions (ins) are given relative to the start of the gene (c). The column designated "Effect" indicates the amino acid level changes that occur as a result of the variant mutation. NA, not applicable; PUF, protein unknown function. # changes codon usage, possibly increasing translation.

Locus tag	Replicon	Position	Annotation	Gene	Predicted Product/Function [COG]	Variant	Effect
			TDA production				
PGA2_c15540	Chromosome	1713355	frameshift_variant	*paaZ2*	Enoyl-CoA hydratase family protein [C]	c.43delC	p.Gln15fs
			DNA replication and repair				
PGA2_c00030	Chromosome	4075	missense_variant	*recF*	DNA replication and repair protein [L]	c.1019C>T	p.Pro340Leu
PGA2_c00030	Chromosome	4088	missense_variant	*recF*	DNA replication and repair protein [L]	c.1032A>T	p.Leu344Phe
PGA2_c11620	Chromosome	1279598	frameshift_variant	PGA2_ c11620	Resolvase [L]	c.1322delT	p.Phe441fs
PGA2_c28190	Chromosome	3101863	frameshift_variant	PGA2_ c28190	Conserved DNA binding protein, YbaB/EbfC family [R]	c.282dupG	p.Lys95fs
			Chemotaxis, transport and metabolism				
PGA2_c14010	Chromosome	1545069	frameshift_variant		Methyl-accepting chemotaxis protein (nitrate/nitrite	c.18delT	p.Phe6fs

					sensing) [N] [T]		
PGA2_c14720	Chromosome	1625957	frameshift_variant	*trkA*	Trk system potassium uptake protein [P]	c.1356insG	p.Ser453fs
PGA2_c14720	Chromosome	1626128	frameshift_variant	*trkA*	Trk system potassium uptake protein [P]	c.1533insT	p.Thr512fs
PGA2_c00120	Chromosome	13457	missense_variant	*gabD1*	Succinate-semialdehyde dehydrogenase [C]	c.352G>T	p.Gly118Trp
EPS production							
PGA2_71p310	pPGA2_71	45578	synonymous_variant	*gmd*	GDP-mannose 4,6-dehydratase [M]	c.1072C>T	p.Leu358Leu
PGA2_71p310	pPGA2_71	45678	synonymous_variant	#*gmd*	GDP-mannose 4,6-dehydratase [M]	c.972C>A	p.Thr324Thr
PGA2_71p310	pPGA2_71	45687	synonymous_variant	#*gmd*	GDP-mannose 4,6-dehydratase [M]	c.963A>G	p.Gly321Gly
PGA2_c09570	Chromosome	1051860	synonymous_variant	*ftsA*	Cell division protein [D]	c.594A>G	p.Gln198Gln
Other							
PGA2_c02240	Chromosome	254279	frameshift_variant		Glyoxylase, beta-lactamase superfamily II- like protein [R]	c.129dupT	p.Leu44fs
PGA2_239p1450	pPGA2_239	159947	upstream_gene_variant		Hypothetical protein [O]	c.-1delC	Unknown
PGA2_239p1120	pPGA2_239	124122	frameshift_variant		Hypothetical protein [S]	c.2506dupG	p.Glu836fs
PGA2_239p1120	pPGA2_239	124163	frameshift_variant		Hypothetical protein [S]	c.2465dupA	p.Asp822fs
PGA2_239p1130	pPGA2_239	128236	missense_variant		Hypothetical protein [S]	c.806C>T	p.Thr269Met
PGA2_95p160	pPGA2_95	24139	upstream_gene_variant		PUF [S]	c.-1_-1insA	Unknown
PGA2_95p160	pPGA2_95	24150	upstream_gene_variant		PUF [S]	c.-1_-1insT	Unknown
PGA2_95p710	pPGA2_95	94382	upstream_gene_variant		Putative plasmid partition protein A [N]	c.-1_-1insT	Unknown
